# Correlations without causation do not support claims of human–LLM reasoning alignment

**DOI:** 10.1073/pnas.2536362123

**Published:** 2026-03-16

**Authors:** Ivan I. Vankov, Federico Adolfi, Rachel F. Heaton, Guillermo Puebla, Jeffrey S. Bowers

**Affiliations:** ^a^Institute of Neurobiology, Bulgarian Academy of Sciences, Sofia 1113, Bulgaria; ^b^Iris.ai, Sofia 1000, Bulgaria; ^c^Ernst Strüngmann Institute for Neuroscience, Max Planck Society, Frankfurt 60528, Germany; ^d^School of Art & Design, University of Illinois Urbana-Champaign, Urbana, IL 61801; ^e^Facultad de Administración y Economía, Universidad de Tarapacá, Santiago 1000000, Chile; ^f^School of Psychology and Neuroscience, University of Bristol, Bristol BS8-1TU, United Kingdom

de Varda et al. (1) ask whether large language reasoning models (LRMs) can serve as models of human reasoning. To address this question, they compared LRM and human performance across seven tasks that varied in difficulty and found that the number of “reasoning” tokens generated by LRMs predicted human reaction times. The authors concluded that “LRMs exhibit strong alignment with human reasoning behavior, not only in terms of which problems are solved correctly but also in the processing cost associated with solving them.” They also took these findings to challenge the view that symbolic architectures are required to support human-like thinking.

**Fig. 1.**
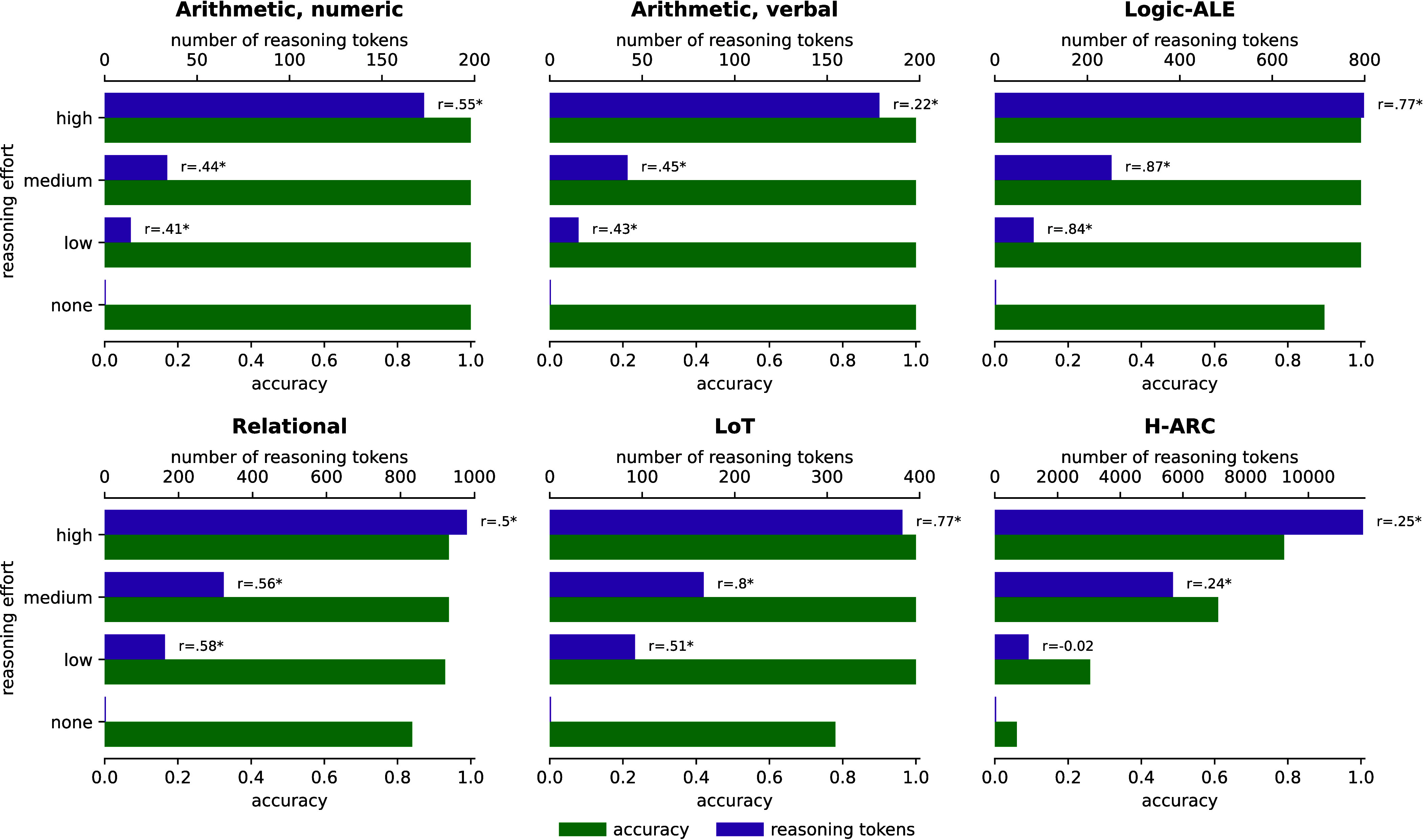
Accuracy and reasoning token counts for gpt-oss-120b under five reasoning-effort conditions. The “none” condition removes the reasoning trace entirely; the low, medium, and high conditions rely on the model training to generate reasoning traces with varying length. Purple bars show the mean number of reasoning tokens; green bars show accuracy. The labels next to the purple bars show the correlation between the number of tokens and human response times taken from the original paper (1).

**Fig. 2.**
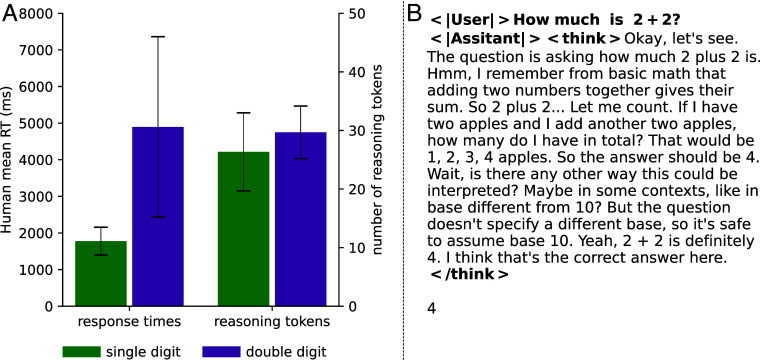
(*Left*) Mean human RTs and number of reasoning traces to 12 Single- and double-digit numeric arithmetic problems taken from de Varda et al. (1). The increased cognitive costs for humans in the double-digit condition are not mirrored in gpt-oss-120b, again highlighting a lack of human-LRM alignment. (*Right*) An example of a reasoning trace generated by Qwen3-32B.

Central to their argument is the assumption that the correlations were causal, with successful predictions reflecting similar reasoning processes. We tested this assumption by manipulating the length of the reasoning trace generated by the LRM gpt-oss-120b on six tasks used by de Varda et al. These manipulations had minimal effect on accuracy in 5 of 6 tasks (Fig. 1). Furthermore, the LRM generated a similar number of tokens when adding single- and double-digit numbers (Fig. 2*A*). If the observed correlations reflected LRM-Human alignment, then increasing/decreasing the number of tokens output by the LRM should have improved/impaired its performance, and the LRM should have generated many more tokens when adding 2-digit numbers. An inspection of a sample LRM reasoning trace illustrates the problem (Fig. 2*B*).

Our results align with studies showing that reasoning-trace length is often unrelated to model performance (2, 3). Why do de Varda et al. observe correlations between token length and RTs? LRMs have been trained on datasets where more difficult problems are paired with longer demonstrations. Accordingly, they may simply be reproducing training-induced output patterns rather than allocating additional computation in response to difficulty.

With regard to de Varda et al.’s claim regarding symbolic architectures, it is important to note that there is a debate as to whether LRMs implement symbolic processes (4, 5). So even if the authors’ premise was correct and LRM-human reasoning was aligned, it would not speak to the role of symbols in thinking.

The field of NeuroAI contains many cases in which correlations are interpreted causally (6). For example, the LLM Centaur was claimed to provide “…tremendous potential for guiding the development of cognitive theories” (7). However, the model failed spectacularly when alignment was assessed through manipulations, such as perfectly repeating 256 digits in a digit-span task (8). Similarly, Brain-Score is designed to “score any ANN on how similar it is to the brain mechanisms for “core object recognition” (9). However, following an image manipulation, it was found that predictions were largely driven by the backgrounds rather than the objects themselves (10). These and many other examples highlight the importance of manipulating variables to test causal hypotheses rather than relying on correlations to draw conclusions regarding alignment.
